# 3-(2-Eth­oxy-2-oxoeth­yl)-4,5,6,7,8,9-hexa­hydrocyclo­octa­[*d*][1,2,3]selena­diazol-3-ium bromide

**DOI:** 10.1107/S2414314625001439

**Published:** 2025-02-28

**Authors:** Dieter Schollmeyer, Heiner Detert

**Affiliations:** a University of Mainz, Department of Chemistry, Duesbergweg 10-14, 55099 Mainz, Germany; Goethe-Universität Frankfurt, Germany

**Keywords:** crystal structure, selenium, heterocycle, medium-sized ring

## Abstract

The title compound, C_12_H_19_N_2_O_2_Se^+^·Br^−^, features a selena­diazole five-membered ring attached to a cyclo­octene ring. A bromine anion is located in the vicinity of the selenium atom [3.0197 (5) Å]

## Abstract 2

The title compound features a selena­diazole five-membered ring attached to a cyclo­octene ring. A bromine anion is located in the vicinity of the selenium atom [3.0197 (5) Å].

## Graphical abstract

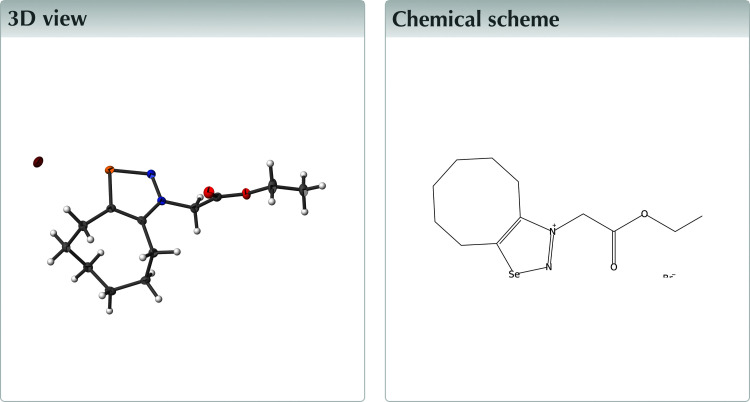

## Structure description

1,2,3-Selena­diazo­les are known as precursors for alkynes, especially strained cyclo­alkynes (Bissinger *et al.*, 1988 ▸; Detert & Meier, 1997 ▸). Jaffari *et al.* (1970 ▸) reported benzo-annulated selena­diazo­lium salts, and the first 1,2,3-selena­diazo­lium salt was described by Butler & Fox (2001 ▸). Recently, *N*-methyl­ated selena­diazo­les were characterized by us (Schollmeyer & Detert, 2016 ▸, 2017 ▸).

**Figure 1:**
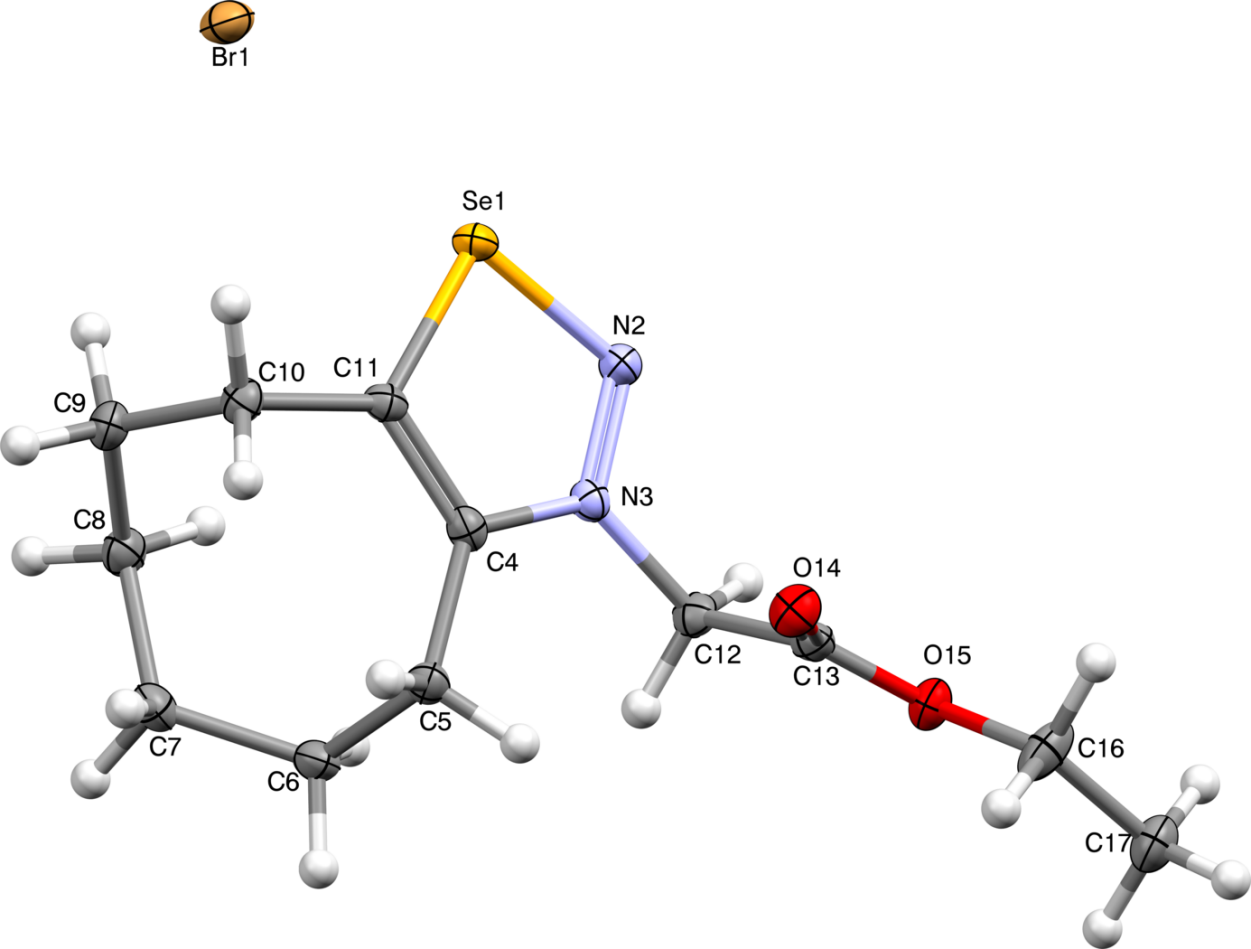
View (Macrae *et al.*, 2020 ▸) of the title compound. Displacement ellipsoids are drawn at the 50% probability level.

**Figure 2:**
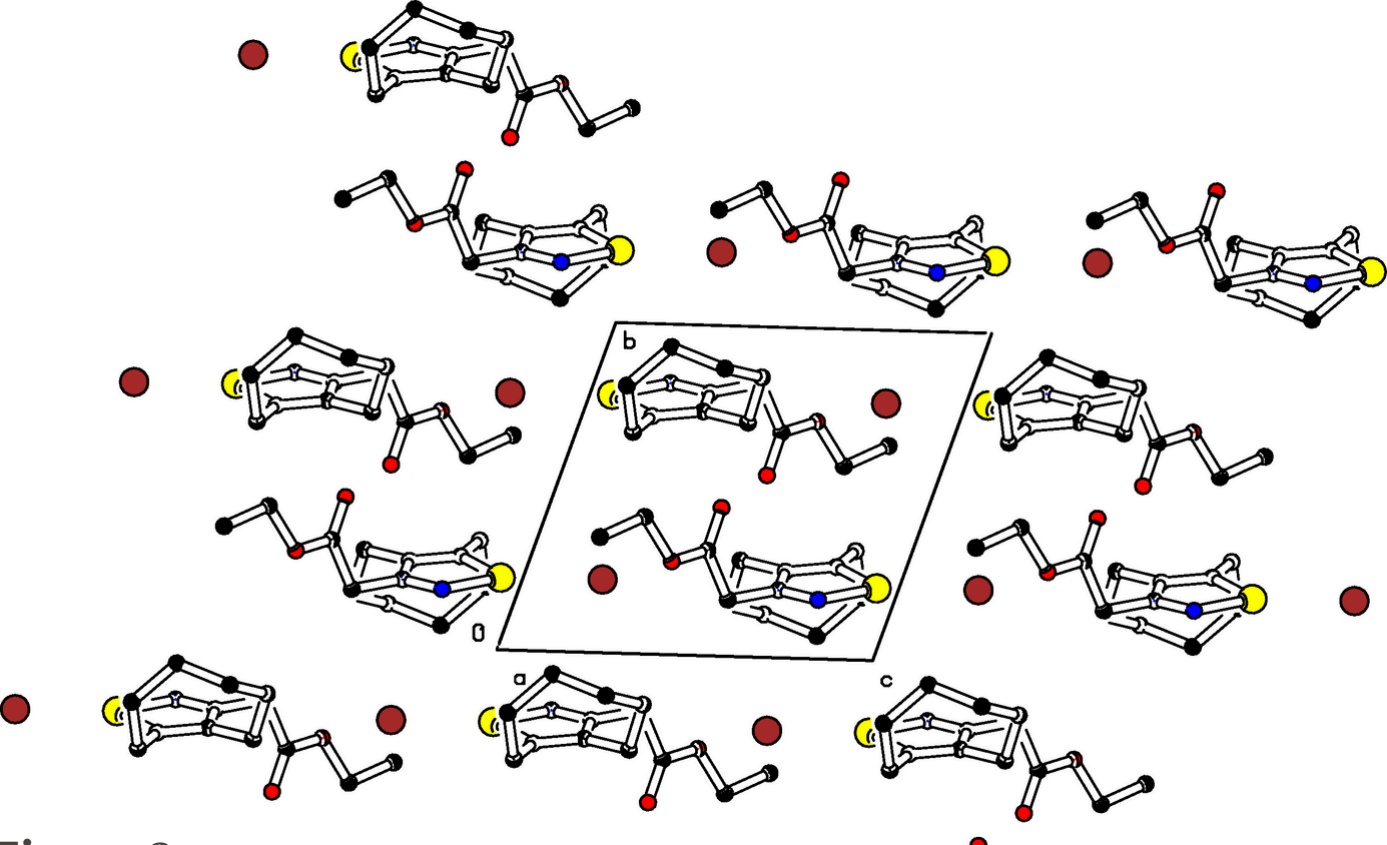
Part of the packing diagram. View along *a*-axis direction (Spek, 2020 ▸).

The mol­ecular structure of the title compound (Fig. 1 ▸) is composed of a cyclo­octene ring with a boat-twist conformation, a 1,2,3-selena­diazole ring, an ethyl­acetate unit, and a bromide anion in the vicinity of the selenium atom. The selena­diazole ring is planar with a maximum deviation of 0.018 (3) Å from the mean plane at N2 whereas N3 is slightly below the ring. In spite of the conformational freedom, the ester unit, C12–C17, is almost planar; here the maximum deviation from the mean plane is 0.117 (2) Å at O15. These planes subtend a dihedral angle of 77.24 (14)°. The cyclo­octene ring adopts a distorted boat-chair conformation (Evans & Boeyens, 1988 ▸). The bromide ion is located in the vicinity of the selenium atom [3.0197 (5) Å], opposite to the carbonyl group and slightly below the selena­diazole plane [0.5364 (3) Å]. The packing is shown in Fig. 2 ▸.

## Synthesis and crystallization

The title compound was prepared by adding ethyl bromo­acetate (2.5 ml) to a solution of cyclo­octeno-1,2,3-selena­diazole (0.9 g, 4 mmol) (Meier & Voigt, 1972 ▸) in nitro­methane (12 ml). The mixture was kept for one month at room temperature under exclusion of light. Two isomeric selena­diazo­lium salts in a 2.5:1 ratio were formed (^1^H-NMR), the main isomer was isolated by evaporation of the solvent and chromatography on silica gel using chloro­form/propanol-2 as eluent. Yield: 0.65 g of the pure title compound (43%), m.p.: 435 K. IR (KBr): 2975, 2912, 2855, 1733, 1711, 1522, 1472, 1447, 1368, 1339, 1240, 1220, 1022 cm^−1^.^1^H-NMR (CDCl_3_, 400 MHz): 5.63 (*s*, 2 H, N—CH_2_; ^13^C-satellites, *J* = 148 Hz), 4.26, (*q*, *J* = 7.5 Hz, OCH_2_), 3.75 (pseudo-*t*, 2 H, 10-CH_2_), 3.17 (pseudo-*t*, 2 H, 5-CH_2_), 1.90 (*qui*, 2 H, 9-CH_2_), 1.78 (*qui*, 2 H, 6-CH_2_), 1.40 (*m*, 4 H, CH_2_), 1.26 ppm (*t*, 3 H, CH_3_). NOE: Irradiation into 5.63: positive NOE at 3.17, 1.78 ppm. 175.6 (C11, Se-satellites, ^1^*J*_C–Se_ = 160 Hz), 164.0 (C=O), 154.4 (C-4) 64.4 (OCH_2_), 61.0 (NCH_2_), 31.2 (C-9), 30.5 (C-10), 28.2 (C-6), 26.8 (C-5), 25.6 (C-7), 24.8 (C-7), 13.9 (CH_3_) ppm. Numbering of atoms according to scheme 1. MS: (EI): 685 (19%, Se_2_Br-isotope pattern), (C_12_H_19_O_2_N_2_Se)_2_Br^+^); 381 (4%, *M*+).

## Refinement

Crystal data, data collection and structure refinement details are summarized in Table 1 ▸.

## Supplementary Material

Crystal structure: contains datablock(s) I, global. DOI: 10.1107/S2414314625001439/bt4164sup1.cif

Structure factors: contains datablock(s) I. DOI: 10.1107/S2414314625001439/bt4164Isup2.hkl

Supporting information file. DOI: 10.1107/S2414314625001439/bt4164Isup3.cml

CCDC reference: 2424352

Additional supporting information:  crystallographic information; 3D view; checkCIF report

## Figures and Tables

**Table 1:** Experimental details

Crystal data	
Chemical formula	C_12_H_19_N_2_O_2_Se^+^·Br^−^
*M*_r_	382.16
Crystal system, space group	Triclinic, *P* 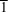
Temperature (K)	120
*a*, *b*, *c* (Å)	8.4924 (7), 9.3927 (7), 9.7799 (8)
α, β, γ (°)	71.379 (6), 86.439 (7), 74.119 (6)
*V* (Å^3^)	710.78 (10)
*Z*	2
Radiation type	Mo *K*α
μ (mm^−1^)	5.45
Crystal size (mm)	0.37 × 0.35 × 0.18

Data collection	
Diffractometer	Stoe *IPDS* 2T
Absorption correction	Integration [*X-RED32* (Stoe, & Cie, 2020 ▸), absorption correction by Gaussian integration, analogous to Coppens (1970 ▸)]
*T*_min_, *T*_max_	0.163, 0.405
No. of measured, independent and observed [*I* > 2σ(*I*)] reflections	6548, 3370, 3085
*R*_int_	0.030
(sin θ/λ)_max_ (Å^−1^)	0.659

Refinement	
*R*[*F*^2^ > 2σ(*F*^2^)], *wR*(*F*^2^), *S*	0.035, 0.096, 1.10
No. of reflections	3370
No. of parameters	164
H-atom treatment	H-atom parameters constrained
Δρ_max_, Δρ_min_ (e Å^−3^)	1.39, −0.84

Computer programs: *X-AREA WinXpose*, *Recipe*, *Integrate* (Stoe & Cie, 2020 ▸), *SHELXT2014* (Sheldrick, 2015*a* ▸), *SHELXL2019/3* (Sheldrick, 2015*b* ▸), *PLATON* (Spek, 2020 ▸) and *Mercury* (Macrae *et al.*, 2020 ▸).

## full crystallographic data

### 3-(2-Ethoxy-2-oxoethyl)-4,5,6,7,8,9-hexahydrocycloocta[d][1,2,3]selenadiazol-3-ium bromide Crystal data

**Table:**

C_12_H_19_N_2_O_2_Se^+^·Br^−^	*F*(000) = 380
*M**_r_* = 382.16	*D*_x_ = 1.786 Mg m^−^^3^
Triclinic, *P*1	Melting point: 435 K
*a* = 8.4924 (7) Å	Mo *K*α radiation, λ = 0.71073 Å
*b* = 9.3927 (7) Å	Cell parameters from 10409 reflections
*c* = 9.7799 (8) Å	θ = 2.5–28.4°
α = 71.379 (6)°	µ = 5.45 mm^−^^1^
β = 86.439 (7)°	*T* = 120 K
γ = 74.119 (6)°	Block, colorless
*V* = 710.78 (10) Å^3^	0.37 × 0.35 × 0.18 mm
*Z* = 2	

### 3-(2-Ethoxy-2-oxoethyl)-4,5,6,7,8,9-hexahydrocycloocta[d][1,2,3]selenadiazol-3-ium bromide Data collection

**Table:**

Stoe IPDS 2T diffractometer	3370 independent reflections
Radiation source: sealed X-ray tube, 12x0.4mm long-fine focus	3085 reflections with *I* > 2σ(*I*)
Detector resolution: 6.67 pixels mm^-1^	*R*_int_ = 0.030
rotation method, ω scans	θ_max_ = 27.9°, θ_min_ = 2.5°
Absorption correction: integration [X-Red32 (Stoe, & Cie, 2020), absorption correction by Gaussian integration, analogous to Coppens (1970)]	*h* = −9→11
*T*_min_ = 0.163, *T*_max_ = 0.405	*k* = −12→12
6548 measured reflections	*l* = −12→12

### 3-(2-Ethoxy-2-oxoethyl)-4,5,6,7,8,9-hexahydrocycloocta[d][1,2,3]selenadiazol-3-ium bromide Refinement

**Table:**

Refinement on *F*^2^	Primary atom site location: dual
Least-squares matrix: full	Hydrogen site location: inferred from neighbouring sites
*R*[*F*^2^ > 2σ(*F*^2^)] = 0.035	H-atom parameters constrained
*wR*(*F*^2^) = 0.096	*w* = 1/[σ^2^(*F*_o_^2^) + (0.0549*P*)^2^ + 1.110*P*] where *P* = (*F*_o_^2^ + 2*F*_c_^2^)/3
*S* = 1.10	(Δ/σ)_max_ = 0.001
3370 reflections	Δρ_max_ = 1.39 e Å^−^^3^
164 parameters	Δρ_min_ = −0.84 e Å^−^^3^
0 restraints	

### 3-(2-Ethoxy-2-oxoethyl)-4,5,6,7,8,9-hexahydrocycloocta[d][1,2,3]selenadiazol-3-ium bromide Special details

**Table:**

Geometry. All esds (except the esd in the dihedral angle between two l.s. planes) are estimated using the full covariance matrix. The cell esds are taken into account individually in the estimation of esds in distances, angles and torsion angles; correlations between esds in cell parameters are only used when they are defined by crystal symmetry. An approximate (isotropic) treatment of cell esds is used for estimating esds involving l.s. planes.
Refinement. Hydrogen atoms were refined as riding on their parent atoms with C—H = 0.99 Å for methylene groups and with C—H = 0.98 Å for methyl groups. Isotropic displacement parameters of the H atoms were set to 1.2*U*_eq_(C) or 1.5*U*_eq_(C_methyl_).

### 3-(2-Ethoxy-2-oxoethyl)-4,5,6,7,8,9-hexahydrocycloocta[d][1,2,3]selenadiazol-3-ium bromide Fractional atomic coordinates and isotropic or equivalent isotropic displacement parameters (Å^2^)

**Table:**

	*x*	*y*	*z*	*U*_iso_*/*U*_eq_	
Br1	0.49934 (4)	0.22173 (3)	1.21107 (3)	0.02302 (10)	
Se1	0.34890 (3)	0.21865 (3)	0.94061 (3)	0.01662 (10)	
O14	0.1838 (3)	0.4488 (2)	0.4557 (2)	0.0206 (4)	
O15	0.0967 (3)	0.2826 (2)	0.3758 (2)	0.0178 (4)	
N2	0.2602 (3)	0.1785 (3)	0.7980 (3)	0.0173 (5)	
N3	0.3497 (3)	0.2043 (3)	0.6843 (3)	0.0144 (4)	
C4	0.4827 (3)	0.2615 (3)	0.6848 (3)	0.0143 (5)	
C5	0.5890 (3)	0.2910 (3)	0.5563 (3)	0.0164 (5)	
H5A	0.626019	0.384490	0.548184	0.020*	
H5B	0.522922	0.313715	0.468181	0.020*	
C6	0.7403 (4)	0.1532 (3)	0.5634 (3)	0.0187 (5)	
H6A	0.704205	0.056317	0.590697	0.022*	
H6B	0.785283	0.168197	0.465499	0.022*	
C7	0.8790 (3)	0.1298 (3)	0.6690 (3)	0.0197 (6)	
H7A	0.977802	0.055683	0.648121	0.024*	
H7B	0.905037	0.230735	0.650026	0.024*	
C8	0.8431 (4)	0.0691 (3)	0.8295 (3)	0.0191 (5)	
H8A	0.940889	−0.013424	0.878496	0.023*	
H8B	0.752247	0.019621	0.838569	0.023*	
C9	0.7976 (3)	0.1894 (3)	0.9106 (3)	0.0187 (5)	
H9A	0.893461	0.229792	0.911700	0.022*	
H9B	0.776542	0.135081	1.012018	0.022*	
C10	0.6482 (3)	0.3294 (3)	0.8502 (3)	0.0163 (5)	
H10A	0.614406	0.385169	0.922162	0.020*	
H10B	0.679218	0.402734	0.761909	0.020*	
C11	0.5076 (3)	0.2798 (3)	0.8151 (3)	0.0139 (5)	
C12	0.2965 (3)	0.1714 (3)	0.5601 (3)	0.0167 (5)	
H12A	0.237092	0.089818	0.594280	0.020*	
H12B	0.393413	0.131917	0.507588	0.020*	
C13	0.1851 (3)	0.3189 (3)	0.4589 (3)	0.0161 (5)	
C16	−0.0031 (4)	0.4150 (4)	0.2620 (4)	0.0263 (7)	
H16A	0.064370	0.484369	0.207894	0.032*	
H16B	−0.094754	0.476150	0.304881	0.032*	
C17	−0.0676 (4)	0.3498 (4)	0.1638 (4)	0.0245 (6)	
H17A	−0.131725	0.435342	0.084350	0.037*	
H17B	−0.137472	0.284484	0.217717	0.037*	
H17C	0.024140	0.286559	0.124624	0.037*	

### 3-(2-Ethoxy-2-oxoethyl)-4,5,6,7,8,9-hexahydrocycloocta[d][1,2,3]selenadiazol-3-ium bromide Atomic displacement parameters (Å^2^)

**Table:**

	*U*^11^	*U*^22^	*U*^33^	*U*^12^	*U*^13^	*U*^23^
Br1	0.02640 (17)	0.02727 (17)	0.01503 (16)	−0.00549 (12)	0.00133 (12)	−0.00775 (12)
Se1	0.01565 (15)	0.02022 (16)	0.01349 (16)	−0.00509 (11)	0.00309 (10)	−0.00490 (11)
O14	0.0246 (10)	0.0171 (9)	0.0214 (10)	−0.0071 (8)	−0.0009 (8)	−0.0062 (8)
O15	0.0195 (10)	0.0160 (9)	0.0174 (10)	−0.0055 (8)	−0.0041 (8)	−0.0030 (8)
N2	0.0151 (11)	0.0218 (11)	0.0152 (11)	−0.0053 (9)	0.0007 (9)	−0.0056 (9)
N3	0.0135 (10)	0.0147 (10)	0.0158 (11)	−0.0046 (8)	0.0007 (8)	−0.0050 (8)
C4	0.0124 (11)	0.0122 (11)	0.0166 (12)	−0.0033 (9)	0.0003 (9)	−0.0021 (9)
C5	0.0161 (12)	0.0181 (12)	0.0147 (13)	−0.0068 (10)	0.0014 (10)	−0.0032 (10)
C6	0.0173 (12)	0.0230 (13)	0.0172 (13)	−0.0063 (11)	0.0046 (10)	−0.0081 (11)
C7	0.0139 (12)	0.0205 (13)	0.0232 (15)	−0.0033 (10)	0.0026 (11)	−0.0062 (11)
C8	0.0179 (13)	0.0187 (13)	0.0173 (13)	−0.0028 (10)	−0.0016 (10)	−0.0027 (10)
C9	0.0163 (12)	0.0225 (13)	0.0173 (13)	−0.0046 (10)	−0.0031 (10)	−0.0060 (11)
C10	0.0178 (12)	0.0155 (12)	0.0169 (13)	−0.0060 (10)	−0.0007 (10)	−0.0053 (10)
C11	0.0149 (12)	0.0117 (11)	0.0138 (12)	−0.0025 (9)	0.0022 (9)	−0.0037 (9)
C12	0.0184 (13)	0.0168 (12)	0.0173 (13)	−0.0061 (10)	0.0002 (10)	−0.0074 (10)
C13	0.0170 (12)	0.0194 (12)	0.0145 (12)	−0.0088 (10)	0.0033 (10)	−0.0059 (10)
C16	0.0371 (17)	0.0187 (13)	0.0204 (15)	−0.0025 (12)	−0.0127 (13)	−0.0043 (11)
C17	0.0245 (15)	0.0270 (15)	0.0213 (15)	−0.0055 (12)	−0.0067 (12)	−0.0064 (12)

### 3-(2-Ethoxy-2-oxoethyl)-4,5,6,7,8,9-hexahydrocycloocta[d][1,2,3]selenadiazol-3-ium bromide Geometric parameters (Å, º)

**Table:**

Se1—N2	1.811 (3)	C8—C9	1.533 (4)
Se1—C11	1.850 (3)	C8—H8A	0.9900
O14—C13	1.208 (3)	C8—H8B	0.9900
O15—C13	1.316 (3)	C9—C10	1.541 (4)
O15—C16	1.469 (4)	C9—H9A	0.9900
N2—N3	1.303 (3)	C9—H9B	0.9900
N3—C4	1.378 (3)	C10—C11	1.490 (4)
N3—C12	1.470 (4)	C10—H10A	0.9900
C4—C11	1.375 (4)	C10—H10B	0.9900
C4—C5	1.498 (4)	C12—C13	1.523 (4)
C5—C6	1.541 (4)	C12—H12A	0.9900
C5—H5A	0.9900	C12—H12B	0.9900
C5—H5B	0.9900	C16—C17	1.491 (4)
C6—C7	1.538 (4)	C16—H16A	0.9900
C6—H6A	0.9900	C16—H16B	0.9900
C6—H6B	0.9900	C17—H17A	0.9800
C7—C8	1.532 (4)	C17—H17B	0.9800
C7—H7A	0.9900	C17—H17C	0.9800
C7—H7B	0.9900		
			
N2—Se1—C11	89.04 (12)	C10—C9—H9A	108.2
C13—O15—C16	115.5 (2)	C8—C9—H9B	108.2
N3—N2—Se1	109.05 (18)	C10—C9—H9B	108.2
N2—N3—C4	120.2 (2)	H9A—C9—H9B	107.4
N2—N3—C12	115.6 (2)	C11—C10—C9	111.8 (2)
C4—N3—C12	124.2 (2)	C11—C10—H10A	109.3
C11—C4—N3	112.7 (2)	C9—C10—H10A	109.3
C11—C4—C5	125.8 (2)	C11—C10—H10B	109.3
N3—C4—C5	121.5 (2)	C9—C10—H10B	109.3
C4—C5—C6	113.5 (2)	H10A—C10—H10B	107.9
C4—C5—H5A	108.9	C4—C11—C10	124.5 (2)
C6—C5—H5A	108.9	C4—C11—Se1	109.0 (2)
C4—C5—H5B	108.9	C10—C11—Se1	126.3 (2)
C6—C5—H5B	108.9	N3—C12—C13	110.1 (2)
H5A—C5—H5B	107.7	N3—C12—H12A	109.6
C7—C6—C5	115.8 (2)	C13—C12—H12A	109.6
C7—C6—H6A	108.3	N3—C12—H12B	109.6
C5—C6—H6A	108.3	C13—C12—H12B	109.6
C7—C6—H6B	108.3	H12A—C12—H12B	108.2
C5—C6—H6B	108.3	O14—C13—O15	126.4 (3)
H6A—C6—H6B	107.4	O14—C13—C12	123.5 (3)
C8—C7—C6	115.6 (2)	O15—C13—C12	110.1 (2)
C8—C7—H7A	108.4	O15—C16—C17	107.3 (2)
C6—C7—H7A	108.4	O15—C16—H16A	110.3
C8—C7—H7B	108.4	C17—C16—H16A	110.3
C6—C7—H7B	108.4	O15—C16—H16B	110.3
H7A—C7—H7B	107.4	C17—C16—H16B	110.3
C7—C8—C9	116.7 (2)	H16A—C16—H16B	108.5
C7—C8—H8A	108.1	C16—C17—H17A	109.5
C9—C8—H8A	108.1	C16—C17—H17B	109.5
C7—C8—H8B	108.1	H17A—C17—H17B	109.5
C9—C8—H8B	108.1	C16—C17—H17C	109.5
H8A—C8—H8B	107.3	H17A—C17—H17C	109.5
C8—C9—C10	116.3 (2)	H17B—C17—H17C	109.5
C8—C9—H9A	108.2		
			
C11—Se1—N2—N3	2.47 (19)	C5—C4—C11—C10	−1.3 (4)
Se1—N2—N3—C4	−3.2 (3)	N3—C4—C11—Se1	0.0 (3)
Se1—N2—N3—C12	177.90 (18)	C5—C4—C11—Se1	−177.0 (2)
N2—N3—C4—C11	2.2 (4)	C9—C10—C11—C4	−87.9 (3)
C12—N3—C4—C11	−179.0 (2)	C9—C10—C11—Se1	87.2 (3)
N2—N3—C4—C5	179.4 (2)	N2—Se1—C11—C4	−1.4 (2)
C12—N3—C4—C5	−1.8 (4)	N2—Se1—C11—C10	−177.1 (2)
C11—C4—C5—C6	82.8 (3)	N2—N3—C12—C13	93.4 (3)
N3—C4—C5—C6	−94.1 (3)	C4—N3—C12—C13	−85.5 (3)
C4—C5—C6—C7	−74.3 (3)	C16—O15—C13—O14	4.8 (4)
C5—C6—C7—C8	71.2 (3)	C16—O15—C13—C12	−173.6 (2)
C6—C7—C8—C9	−102.0 (3)	N3—C12—C13—O14	21.8 (4)
C7—C8—C9—C10	57.4 (3)	N3—C12—C13—O15	−159.8 (2)
C8—C9—C10—C11	45.7 (3)	C13—O15—C16—C17	169.4 (3)
N3—C4—C11—C10	175.8 (2)		

## References

[bb1] Bissinger, H.-J., Detert, H. & Meier, H. (1988). *Liebigs Ann. Chem.* pp. 221–224.

[bb2] Butler, R. N. & Fox, A. (2001). *J. Chem. Soc. Perkin Trans. 1*, pp. 394–397.

[bb3] Coppens, P. (1970). *Crystallographic Computing*, edited by F. R. Ahmed, S. R. Hall & C. P. Huber, pp. 255–270. Copenhagen: Munksgaard.

[bb4] Detert, H. & Meier, H. (1997). *Liebigs Ann. Recl*, pp. 1557–1563.

[bb15] Evans, D. G. & Boeyens, J. C. A. (1988). *Acta Cryst.* B**44**, 663–671.

[bb5] Jaffari, G. A., Nunn, A. J. & Ralph, J. T. (1970). *J. Chem. Soc. C*, pp. 2060–2062.

[bb6] Macrae, C. F., Sovago, I., Cottrell, S. J., Galek, P. T. A., McCabe, P., Pidcock, E., Platings, M., Shields, G. P., Stevens, J. S., Towler, M. & Wood, P. A. (2020). *J. Appl. Cryst.* **53**, 226–235. 10.1107/S1600576719014092 PMC6998782 32047413

[bb7] Meier, H. & Voigt, E. (1972). *Tetrahedron*, **28**, 187–198.

[bb8] Schollmeyer, D. & Detert, H. (2016). *IUCrData*, **1**, x161950.

[bb9] Schollmeyer, D. & Detert, H. (2017). *IUCrData*, **2**, x170167.

[bb10] Sheldrick, G. M. (2015*a*). *Acta Cryst.* A**71**, 3–8.

[bb11] Sheldrick, G. M. (2015*b*). *Acta Cryst.* C**71**, 3–8.

[bb12] Spek, A. L. (2020). *Acta Cryst.* E**76**, 1–11. 10.1107/S2056989019016244 PMC6944088 31921444

[bb13] Stoe & Cie (2020). *X-RED* and *X-AREA*. Stoe & Cie, Darmstadt, Germany.

